# cGAS activation in classical dendritic cells causes autoimmunity in TREX1-deficient mice

**DOI:** 10.1073/pnas.2411747121

**Published:** 2024-09-10

**Authors:** Tong Li, Seoyun Yum, Junjiao Wu, Minghao Li, Yafang Deng, Lijun Sun, Xiaoxia Zuo, Zhijian J. Chen

**Affiliations:** ^a^Department of Molecular Biology and Center for Inflammation Research, University of Texas Southwestern Medical Center, Dallas, TX 75390; ^b^Department of Rheumatology and Immunology, Xiangya Hospital, Central South University, Changsha, Hunan 410078, China; ^c^National Clinical Research Center for Geriatric Disorders, Xiangya Hospital, Changsha, Hunan 410078, China; ^d^HHMI, Chevy Chase, MD 20815

**Keywords:** cGAS, STING, TREX1, inflammation, dendritic cell

## Abstract

Detection of cytosolic DNA by the cyclic GMP-AMP (cGAMP) synthase (cGAS)–stimulator of interferon genes (STING) pathway provides immune defense against pathogens and cancer but can also cause autoimmunity when overactivated. The exonuclease three prime repair exonuclease 1 (TREX1) degrades DNA in the cytosol and prevents cGAS activation by self-DNA. Loss-of-function mutations of the TREX1 gene are linked to autoimmune diseases such as Aicardi–Goutières syndrome, and mice deficient in TREX1 develop lethal inflammation in a cGAS-dependent manner. In order to determine the type of cells in which cGAS activation drives autoinflammation, we generated conditional cGAS knockout mice on the Trex1^–/–^ background. Here, we show that genetic ablation of the cGAS gene in classical dendritic cells (cDCs), but not in macrophages, was sufficient to rescue Trex1^–/–^ mice from all observed disease phenotypes including lethality, T cell activation, tissue inflammation, and production of antinuclear antibodies and interferon-stimulated genes. These results show that cGAS activation in cDC causes autoinflammation in response to self-DNA accumulated in the absence of TREX1.

## Significance

Mice deficient in three prime repair exonuclease 1 (TREX1) accumulate cytosolic DNA, causing a lethal autoimmune disease that resembles Aicardi–Goutières syndrome in humans. Cyclic GMP-AMP (cGAMP) synthase (cGAS) has been shown to mediate this autoimmunity by detecting cytosolic DNA, and inhibitors targeting the cGAS pathway reduce inflammation and disease phenotypes in Trex1^–/–^ mice. Here, using conditional cGAS knockout mice, we show that deletion of the cGAS gene in classical dendritic cells is sufficient to rescue lethal inflammation in Trex1-deficient mice. These results show that cGAS activation in classical dendritic cells is the major culprit of autoinflammatory diseases caused by Trex1 deficiency, suggesting that targeting cGAS inhibition in classical dendritic cell (cDC) may be sufficient to provide therapeutic benefits.

The cytosolic DNA-sensing pathway protects hosts from pathogens by eliciting appropriate immune responses, but aberrant activation of this pathway can cause autoimmune diseases. Cyclic GMP-AMP (cGAMP) synthase (cGAS) was identified as an immune sensor detecting cytosolic double-stranded DNA that arose from viral or bacterial infections (1, 2). cGAS binds DNA without sequence specificity, allowing it to detect both self and nonself DNA (3–5). Upon DNA binding, cGAS converts ATP and GTP into 2’3’-cGAMP, which binds to and activates the adaptor protein stimulator of interferon genes (STING) (6–8). STING subsequently activates TANK-binding kinase 1 (TBK1) and IκB kinase (IKK) to activate transcription factors interferon regulatory factor 3 (IRF3) and NF-κB, respectively (9, 10). These transcription factors induce interferons (IFNs) and inflammatory cytokines, which elicit antiviral or antitumor immune responses. As cGAS binds to cytosolic DNA regardless of sequence or origin, the pathway is tightly regulated to prevent aberrant activation by endogenous DNA that may be released from the nucleus or mitochondria.

Three prime repair exonuclease 1 (TREX1) degrades DNA in the cytosol and thus is an essential negative regulator of the cytosolic DNA-sensing pathway (11, 12). Loss-of-function mutations in the human Trex1 gene cause autoinflammatory and autoimmune diseases such as Aicardi–Goutières syndrome (AGS), familial chilblain lupus, and retinal vasculopathy with cerebral leukodystrophy (13–16). Trex1 mutations are also associated with the autoimmune disease systemic lupus erythematosus (SLE) (17, 18). These diseases are characterized by multisystem inflammation, excessive expression of interferon-stimulated genes (ISGs), and the production of a variety of autoantibodies, especially antinuclear antibody (ANA). Similar to human patients with Trex1 mutations, Trex1^–/–^ mice develop lethal autoimmunity characterized by type I IFN-dependent inflammation (19, 20). The cGAS–STING pathway has been shown to induce autoimmunity in Trex1^–/–^ mice as the genetic ablation of cGAS or STING rescues mice from disease phenotypes (21–24).

Investigating the origin of type I IFNs during TREX1 deficiency is critical to understanding the disease mechanism and identifying therapeutic targets. Trex1^–/–^ fibroblasts, macrophages, and dendritic cells in culture expressed elevated ISGs, indicating a widespread type I IFN induction by endogenous DNA (21, 25, 26). In vivo, hematopoietic cells were suggested as a source of type I IFNs; TREX1 deficiency in hematopoietic cells was necessary and sufficient to drive autoimmunity in adoptive transfer experiments (26, 27). Supporting this notion, immune cells infiltrating the hearts of Trex1^–/–^ mice were shown as the major source of type I IFN and cytokines in the heart, while selective deletion of Trex1 in cardiomyocytes did not induce inflammatory cytokines (26, 27). Lymphocytes were required for the autoimmune phenotypes but were dispensable for type I IFN induction in Trex1^–/–^ mice, suggesting that lymphocytes are type I IFN responders rather than producers (20, 22). In the Trex1^–/–^ IFNβ reporter mice, IFNβ induction was detected in plasmacytoid dendritic cells (pDCs), classical dendritic cells (cDCs), and macrophages, but not in T or B cells (27). Furthermore, selective loss of the Trex1 gene in Clec9a-expressing cells (28) was sufficient to induce autoimmunity (27), indicating that deletion of Trex1 in cDC1 is sufficient to drive autoimmunity.

To determine the type of immune cells detecting endogenous DNA and causing autoimmunity in Trex1^–/–^ mice, we generated three different conditional cGAS knockout mice on the Trex1^–/–^ background: CD11c^cre+^ cGAS^Flox/Flox^, LysM^cre+^ cGAS^Flox/Flox^, and zDC^cre+^ cGAS^Flox/Flox^ mice with selective deletion of the cGAS gene in macrophages, dendritic cells (both pDC and cDC), and cDC, respectively. Using these conditional knockout (KO) mice, we demonstrate that cGAS activation in cDCs, but not in macrophages, is essential for all observed disease phenotypes of Trex1^–/–^ mice. Our results suggest cDCs as a therapeutic target of cGAS-inhibiting treatments for autoimmune diseases caused by cytosolic self-DNA accumulation.

## Results

### cGAS Deletion in cDCs Rescues the Lethality of Trex1^–/–^ cGAS^Flox/Flox^ Mice.

**Fig. 1.**
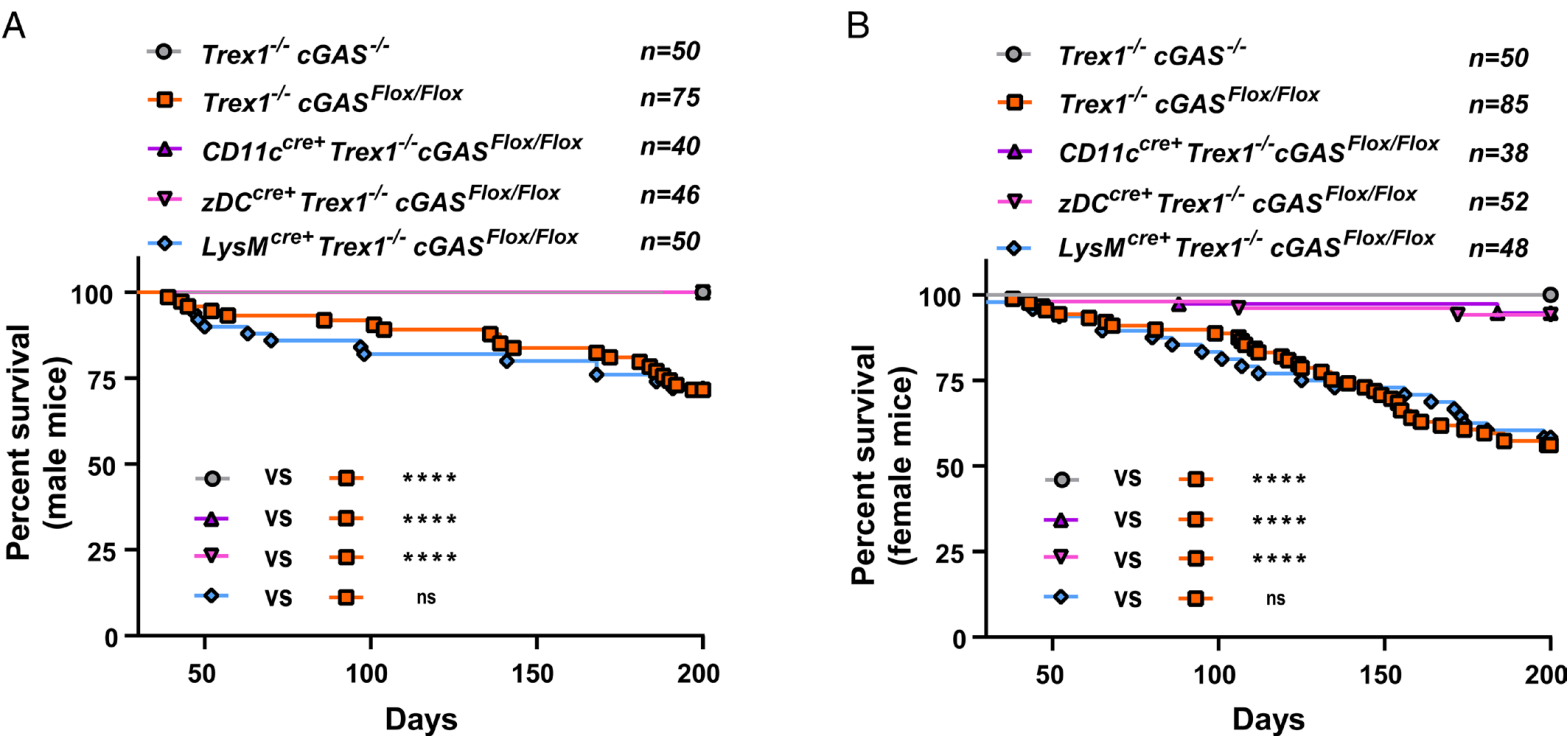
Conditional cGAS knockout in cDCs, but not in macrophages, rescues the lethality of Trex1^–/–^ cGAS^Flox/Flox^ mice. Survival curves of male (*A*) and female (*B*) Trex1^–/–^ mice of indicated genotypes. All mice were on the C57BL/6 background. Statistical analysis was performed using the Mantel–Cox test. ****P < 0.0001.

Trex1^–/–^ mice develop a lethal disease within a few months after birth due to severe inflammation including cardiomyopathy (19). We confirmed that both male and female Trex1^–/–^ cGAS^Flox/Flox^ mice developed significant lethality within 200 d while all Trex1^–/–^ cGAS^–/–^ mice survived (Fig. 1 *A* and *B*). To determine the cell types in which cGAS activation causes lethal inflammation, we generated conditional cGAS KO mice: CD11c^cre+^ cGAS^Flox/Flox^, LysM^cre+^ cGAS^Flox/Flox^, and zDC^cre+^ cGAS^Flox/Flox^ mice on the Trex1^–/–^ background. Western blotting confirmed the reduction of the cGAS protein in splenic DCs from Trex1^–/–^ CD11c^cre+^ cGAS^Flox/Flox^ (Trex1^–/–^ CD11c-cGAS KO) mice compared to their littermate control; cells from the peritoneal cavity that are abundant of macrophages retained the cGAS expression (*SI Appendix*, Fig. S1*A*) (29). In contrast, Trex1^–/–^ LysM^cre+^ cGAS^Flox/Flox^ (Trex1^–/–^ LysM-cGAS KO) mice showed reduced cGAS protein expression in peritoneal cavity cells but not in splenic DCs (*SI Appendix*, Fig. S1*B*). zDC^cre+^ mice express the Cre recombinase in cDCs but not in pDCs (30); Trex1^–/–^ zDC^cre+^ cGAS^Flox/Flox^ (Trex1^–/–^ zDC-cGAS KO) mice had lower cGAS expression in splenic cDCs (CD11c^+^ MHC^high^) but not in pDCs (CD11c^+^ MHC^low^) (*SI Appendix*, Fig. S1*C*). Strikingly, cGAS deletion in CD11c-Cre or zDC-Cre-expressing cells rescued both male and female Trex1^–/–^ mice from lethality (Fig. 1 *A* and *B*). In contrast, Trex1^–/–^ LysM-cGAS KO mice did not show any improvement in the survival rate compared to Trex1^–/–^ cGAS^Flox/Flox^ mice. These results demonstrate that cGAS activation in cDCs is responsible for the lethality of Trex1^–/–^ mice.

### cGAS Activation in cDCs Is Essential for the Elevated ISGs in Trex1^–/–^ Mice.

**Fig. 2.**
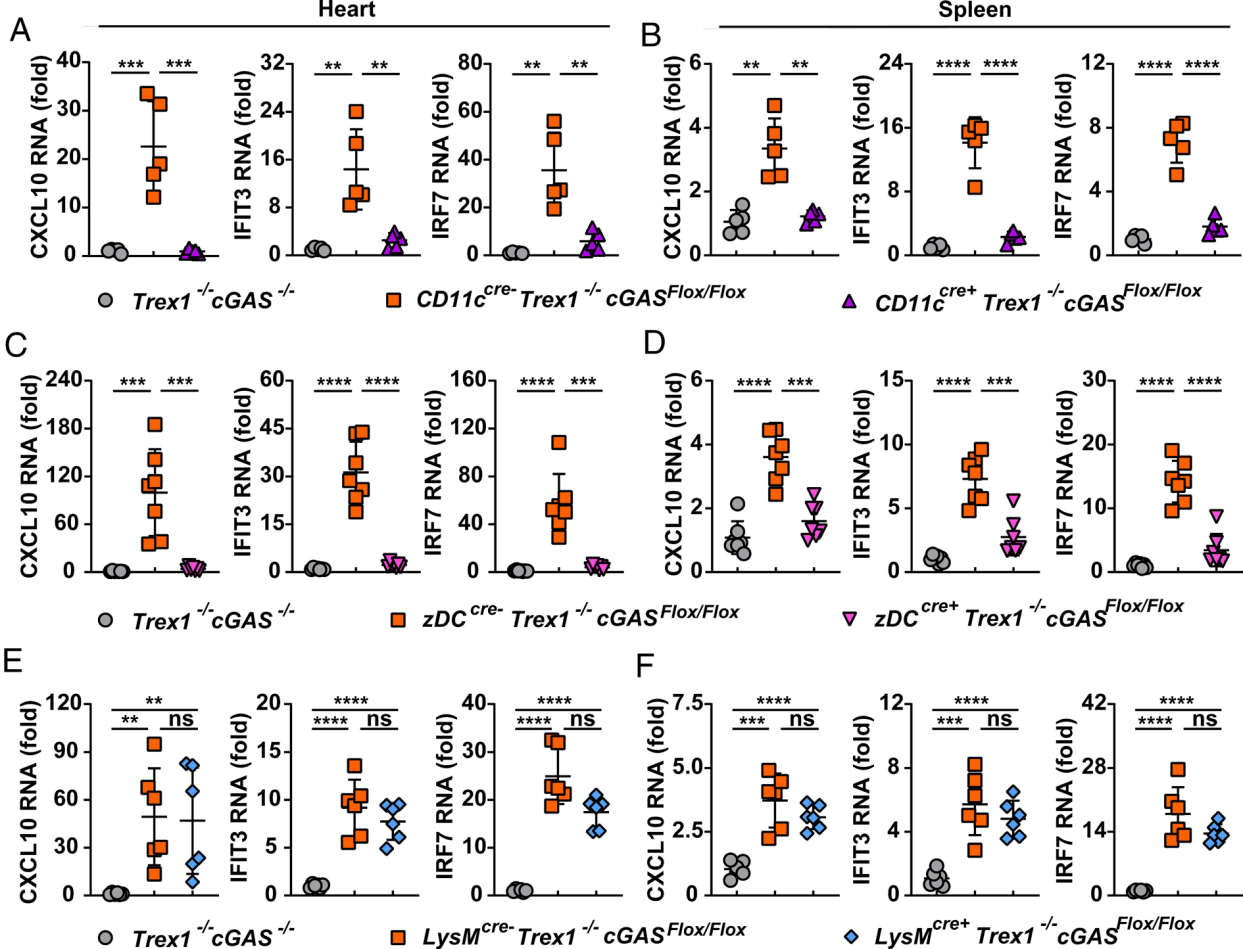
cGAS in cDCs, but not in macrophages, is essential for the expression of ISGs in Trex1^–/–^ cGAS^Flox/Flox^ mice. qRT-PCR analysis of indicated ISGs in the hearts (*A*, *C*, *E*) and spleens (*B*, *D*, *F*) from 3-mo-old mice of indicated genotypes. Fold changes are relative to Trex1^–/–^ cGAS^–/–^ mice. Error bars represent SD. **P < 0.01, ***P < 0.001, ****P < 0.0001.

Similar to AGS patients, Trex1^–/–^ mice express high levels of type I IFNs and ISGs in inflamed tissues. We confirmed the elevated expression of CXCL10, IFIT3, IRF7, ISG15, and IFNγ in the hearts and spleens from all Cre-negative Trex1^–/–^ cGAS^Flox/Flox^ mice (Fig. 2 and *SI Appendix*, Fig. S2). In order to determine the major source of type I IFNs and the subsequent ISG expression, we compared ISG expression in Trex1^–/–^ conditional cGAS KO mice. Both CD11c- and zDC-cGAS KO mice showed dramatically reduced ISG levels in the hearts and spleens compared to their Cre-negative littermates (Fig. 2 *A*–*D* and *SI Appendix*, Fig. S2 *A*–*D*); their ISG levels were comparable to that of Trex1^–/–^ cGAS^–/–^ mice. LysM-cGAS KO mice, however, retained the high level of ISG expression (Fig. 2 *E* and *F* and *SI Appendix*, Fig. S2 *E*–*F*). This result suggests that cDCs are the major source of cGAS-induced type I IFNs during TREX1 deficiency.

### cGAS Activation in cDCs Mediates T Cell Activation and ANA Production in Trex1^–/–^ Mice.

**Fig. 3.**
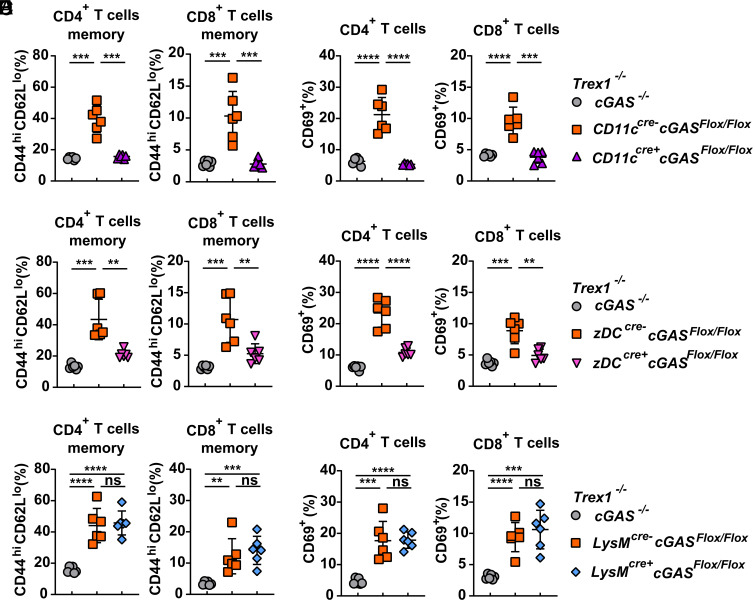
Conditional cGAS knockout in cDCs, but not in macrophages, reduces T cell activation in Trex1^–/–^ cGAS^Flox/Flox^ mice. Flow-cytometric analysis of memory T cells (*A*, *C*, *E*) and CD69^+^ cells (*B*, *D*, *F*) in splenic CD4^+^ and CD8^+^ T cells from 3-mo-old mice of the indicated genotypes. Error bars represent SD. **P < 0.01, ***P < 0.001, ****P < 0.0001.

Chronic expression of type I IFNs is associated with autoimmunity characterized by the production of autoreactive T cells and ANA. Splenocytes from Cre-negative Trex1^–/–^ cGAS^Flox/Flox^ mice displayed elevated levels of Th1 cells as shown by the production of IFNγ upon stimulation with phorbol myristate acetate (PMA) and ionomycin (*SI Appendix*, Fig. S3). Trex1^–/–^ LysM-cGAS KO mice retained the elevated level of Th1 cells in the spleen compared to their Cre-negative littermates, whereas Trex1^–/–^ CD11c- and zDC-cGAS KO mice showed lower numbers of IFNγ-expressing CD4^+^ T cells, similar to Trex1^–/–^ cGAS^–/–^ mice (*SI Appendix*, Fig. S3). In addition, Trex1^–/–^ CD11c- and zDC-cGAS KO mice showed significantly reduced number of memory (CD44^hi^ CD62L^lo^; Fig. 3 *A* and *C*) and activated (CD69^+^, Fig. 3 *B* and *D*) CD4^+^ and CD8^+^ T cells, indicating that cGAS activation in cDCs is essential for activating T cells during TREX1 deficiency. Consistently, the surface level of Ly6c on CD8^+^ T cells was lower in Trex1^–/–^ CD11c- and zDC-cGAS KO mice compared to their Cre-negative littermates (*SI Appendix*, Fig. S4 *A* and *B*). Trex1^–/–^ LysM-cGAS KO mice showed numbers of memory and activated T cells similar to Cre-negative littermates (Fig. 3 *E* and *F* and *SI Appendix*, Fig. S4*C*), indicating that cGAS activation in macrophages is dispensable for T cell activation in Trex1^–/–^ mice.

**Fig. 4.**
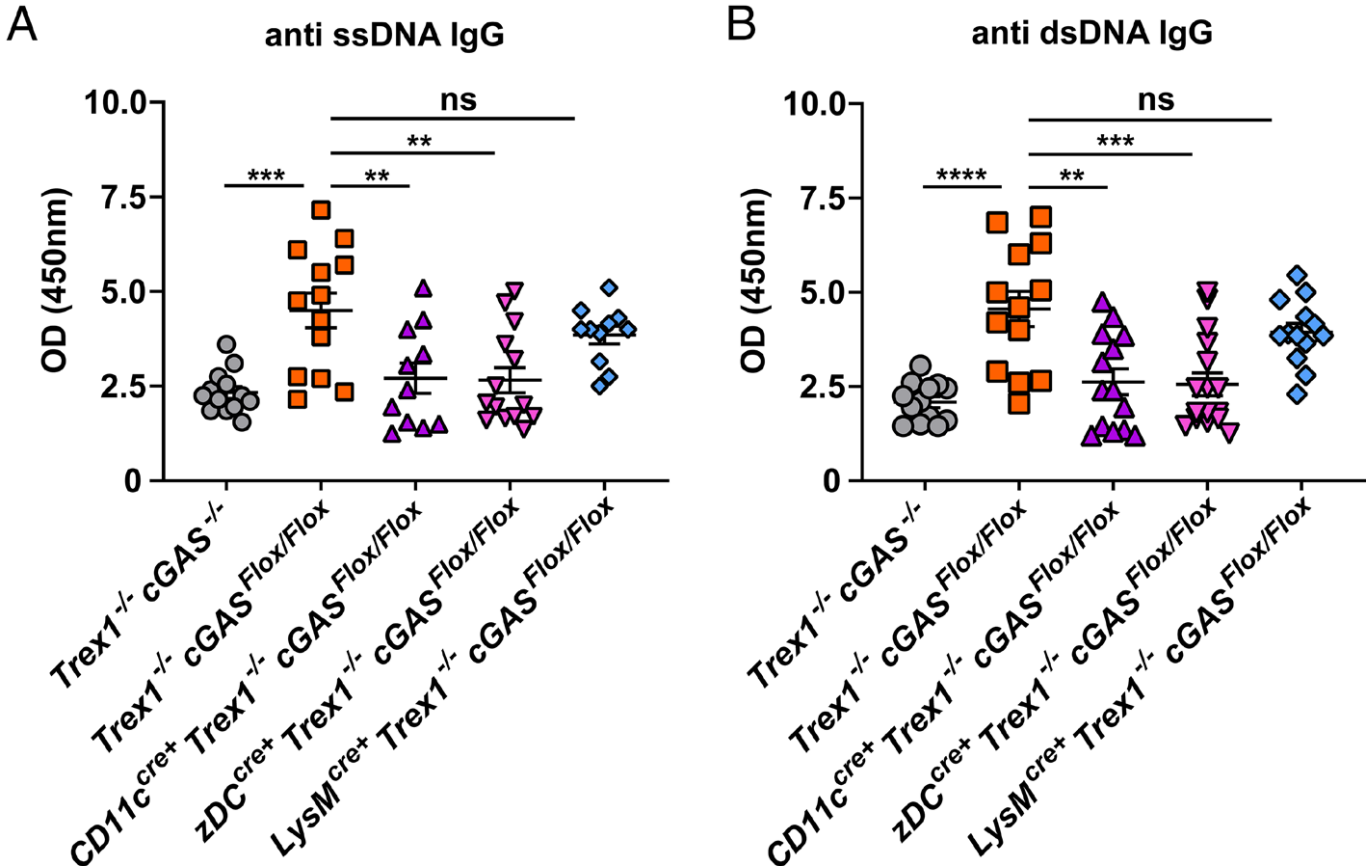
cGAS in cDCs, but not in macrophages, is responsible for the generation of autoantibodies in Trex1^–/–^ cGAS^Flox/Flox^ mice. ELISA of anti-ssDNA (*A*) and anti-dsDNA (*B*) in the serum of 200-d-old mice of the indicated genotypes. Error bars represent SD. **P < 0.01, ***P < 0.001, ****P < 0.0001.

ANA is used as a primary marker for systemic autoimmune diseases such as SLE. In Trex1^–/–^ mice, cGAS activation was responsible for the ANA production (23). We confirmed that Trex1^–/–^ cGAS^Flox/Flox^ mice showed higher levels of anti-ssDNA or anti-dsDNA IgG in their sera compared to Trex1^–/–^ cGAS^–/–^ mice (Fig. 4 *A* and *B*). This ANA level was significantly reduced in Trex1^–/–^ CD11c- and zDC-cGAS KO mice but not in LysM-cGAS KO mice (Fig. 4 *A* and *B*). Altogether, these data demonstrate that cGAS activation in cDCs, but not in macrophages, drives autoimmune phenotypes such as T cell activation and ANA production in Trex1^–/–^ mice.

### cGAS Activation in cDCs Causes Multiorgan Inflammation in Trex1^–/–^ Mice.

**Fig. 5.**
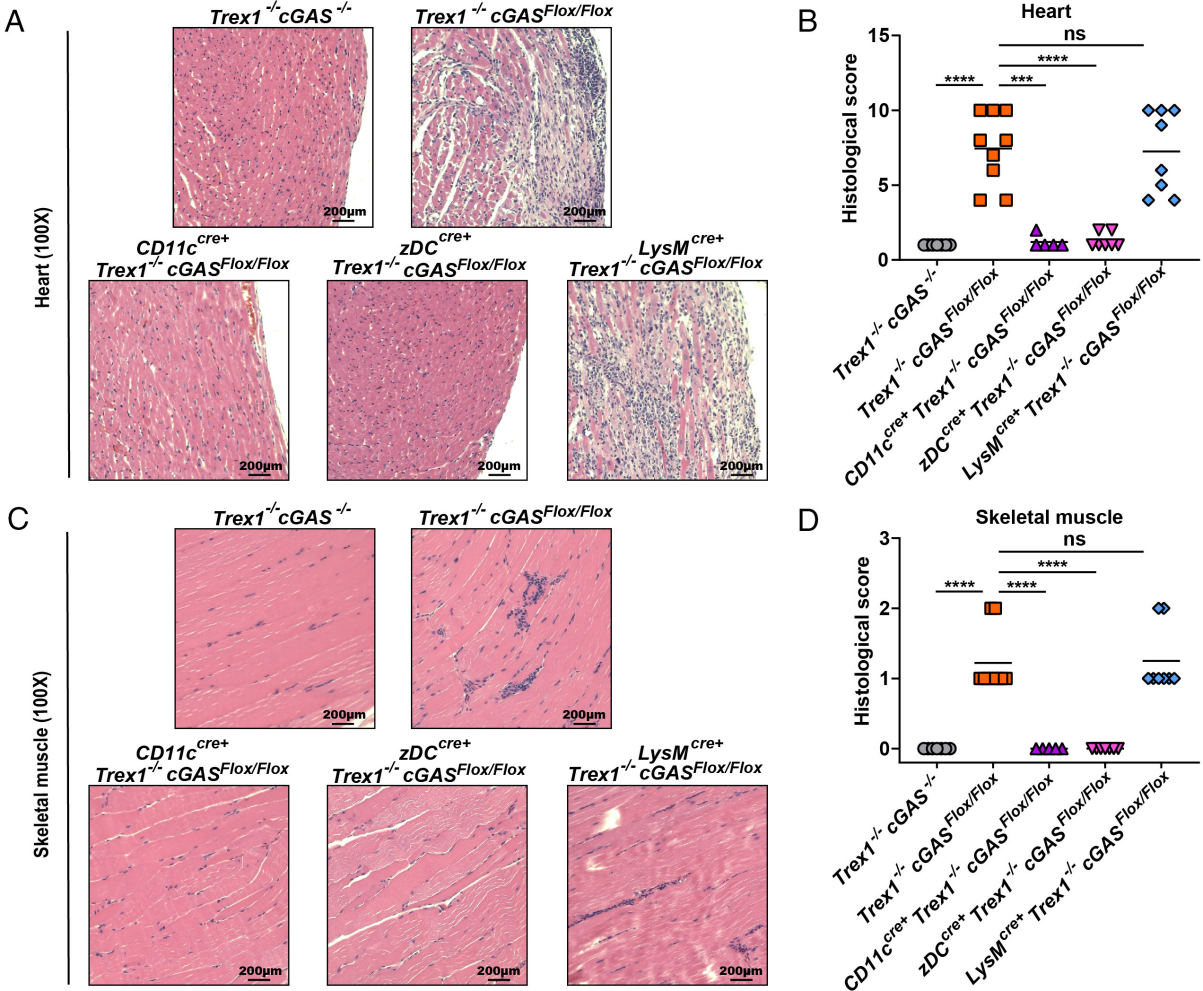
cGAS in cDCs, but not in macrophages, mediates multiorgan inflammation in Trex1^–/–^ cGAS^Flox/Flox^ mice. (*A* and *C*) Representative H&E-stained heart (*A*) and skeletal muscle (*C*) sections from 6-mo-old mice of the indicated genotypes. Blue-stained cells indicate leukocytes infiltrating the heart and skeletal muscle. (*B* and *D*) Blinded analysis of the tissue section. Histological scores were calculated as described in *Materials and Methods*. Statistical analysis was performed with a two-tailed, unpaired Student’s t test. ***P < 0.001, ****P < 0.0001.

Autoimmune diseases are characterized by the loss of self-tolerance and the subsequent severe inflammation and tissue destruction. Trex1^–/–^ LysM-cGAS KO mice retained the inflammatory pathology in the heart, skeletal muscle, and kidney that was also observed in Trex1^–/–^ cGAS^Flox/Flox^ mice (Fig. 5 and *SI Appendix*, Fig. S5). Strikingly, selectively deleting the cGAS gene in CD11c- or zDC-expressing cells rescued Trex1-deficient mice from tissue inflammation; histological scores of the heart, skeletal muscle, and kidney in these mice were comparable to that of Trex1^–/–^ cGAS^–/–^ mice (Fig. 5 and *SI Appendix*, Fig. S5). Inflammation and hyperactivation of immune cells were also indicated by the splenomegaly observed in Trex1^–/–^ LysM-cGAS KO mice that was absent in CD11c- or zDC-cGAS KO mice (*SI Appendix*, Fig. S6 *A*–*C*). Taken together, our results show that cGAS activation in cDCs, but not in macrophages, causes multiorgan inflammation in Trex1^–/–^ mice.

## Discussion

Immune signaling pathways are tightly regulated to maintain self-tolerance and avoid self-tissue destruction. TREX1 regulates the cGAS immune sensing pathway by reducing the cytosolic DNA level; thus, the loss-of-function of TREX1 causes overactivation of cGAS by endogenous DNA and leads to autoimmunity. Despite the widespread expression of cGAS and TREX1 in organs and cells, our study shows that cGAS activation in cDCs is responsible for the disease phenotypes in Trex1^–/–^ mice. Conditional deletion of the cGAS gene in cDCs, but not in macrophages, rescued mice from lethality, ISG production, T cell activation, ANA generation, and tissue inflammation. However, it is still uncertain whether cGAS activation in cDCs causes the Aicardi–Goutieres Syndrome in humans, because human patients with loss-of-function mutations of Trex1 develop severe encephalitis, whereas Trex1^–/–^ mice exhibit myocarditis and inflammation in multiple organs but no apparent encephalitis. More work is needed to determine why Trex1 deficiency in mice and humans affects different organs and in what cell types does cGAS activation cause diseases in humans.

Our data show that cGAS activation in cDCs is necessary to activate T cells and induce ANA in mice during TREX1 deficiency. However, the contribution of subsets of cDCs has yet to be characterized. cDCs are divided into two subsets: cDC1 and cDC2. Although either type of cDCs can activate CD4^+^ and CD8^+^ T cells, cDC1 is specialized in stimulating CD8^+^ T cells, whereas cDC2 stimulates CD4^+^ T cells more efficiently. A previous study reported that the deletion of the Trex1 gene in Clec9a-expressing cells is sufficient to cause autoimmunity (27). Since Clec9a^cre+^ mice delete the floxed gene in ~100% of cDC1 (CD8^+^ cDCs), ~50% of cDC2 (CD11b^+^ cDCs), and ~20% of pDCs (31), these results further support an essential role for DNA-sensing in cDCs. Further investigation on cDC subsets will help understand how cGAS activation in these cells contributes to the production of autoreactive T cells and ANA.

We demonstrated that cDCs drive disease phenotypes during TREX1 deficiency in this study, but pDCs were also suggested to contribute to other autoimmune diseases such as SLE. Higher numbers of pDCs were recruited to inflamed SLE patient tissues, and the transient depletion of pDCs reduced the disease phenotype of a mouse lupus model (32, 33). pDCs are specialized for the production of type I IFNs via Toll-like receptor (TLR) 7 and TLR9, which sense RNA and unmethylated DNA in the endosomes, respectively. pDCs were shown to induce type I IFNs in response to dsDNA virus infection through TLR9, while cDCs and monocyte-derived DCs responded to DNA in a cGAS-dependent manner (34–36). Similar to the case of virus infection, pDCs may contribute to TLR9-mediated inflammation in autoimmune diseases, whereas cDCs mainly contribute to cGAS-mediated inflammation. SLE is a disease with various genetic defects that lead to the activation of TLR, cGAS, and other pathways (37–39). Activation of the cGAS pathway was detected in some SLE patients (40), and apoptosis-derived membrane vesicles from SLE patients activated the STING pathway (41). As specific immune pathways are activated in different types of DCs, identifying the overactive immune signaling pathways in autoimmune patients may allow us to understand the type of immune cells contributing to disease phenotypes.

cGAS activation in macrophages has the potential to contribute to autoinflammation by activating T cells and by producing inflammatory cytokines. During myocardial infarction, massive cell death activated the cGAS–STING pathway in macrophages, worsening the survival of mice (42, 43). In the deoxyribonuclease II (DNase II)-deficient autoimmune model, apoptotic bodies accumulated in macrophages, which activated the cGAS pathway to produce IFNs and cause lethal anemia (44–46). The defect of clearing apoptotic cells was also proposed to be one of the causes of SLE (47). In contrast, our study shows that cGAS in macrophages was dispensable for autoimmunity caused by TREX1 deficiency. Since TREX1 degrades DNA in the cytosol whereas DNase II degrades DNA in the lysosome, the type of cells driving autoimmunity via cGAS activation may depend on the subcellular localization of self-DNA. Further research on DNA localization and the immune cells responsible for other autoimmune diseases will enrich our understanding of the initiation of autoimmunity.

In summary, our study identified cDCs as the major driver of autoimmunity in Trex1^-/–^ mice. Currently, no specific treatment is available for autoimmune diseases with TREX1 mutations. Due to its essential role in the pathogenesis, the cGAS–STING pathway has been suggested as a new therapeutic target for autoimmune diseases caused by cytosolic DNA accumulation. Deletion of one allele of the cGAS gene (cGas^+/−^) dramatically improved the morbidity of Trex1^–/–^ mice, suggesting that partial inhibition of the cGAS pathway may be sufficient to provide therapeutic benefits (23, 24). Several inhibitors targeting cGAS (48) or STING (49, 50) have been reported to reduce ISG expression and alleviate the disease phenotypes of Trex1^–/–^ mice. Our study suggests that inhibition of cGAS in one specific cell type, cDCs, will be sufficient to ameliorate autoimmune diseases caused by Trex1 deficiency. Further understanding the origin of type I IFNs in other interferonopathies will also help design targeted therapeutic strategies for a variety of autoimmune diseases.

## Materials and Methods

### Mouse Strains and Genotyping.

Mouse strains utilized in these experiments were based on the C57BL/6 strain. Trex1^+/−^ mice were provided by Dr. Nan Yan (University of Texas Southwestern Medical Center) (23). CD11c^cre+^ and zDC^cre+^ mice were provided by Dr. Yangxin Fu (University of Texas Southwestern Medical Center) (51). LysM^cre+^ mice were purchased from The Jackson Laboratory. An in vitro fertilization process was utilized to create cGAS^Flox/Flox^ mice. The targeting vector contained the Neomycin cassette flanked by flippase recombinase target sites and the exon 2 of cGAS gene flanked by LoxP sites; this vector was inserted at the position 784379955 of Chromosome 9, upstream of the “critical” exon of cGAS. Mice carrying the targeting vector sequence were crossed with transgenic mice carrying flippase recombinase to remove the selection cassette, generating cGAS^Flox/Flox^ mice. Mice were genotyped from toe or tail genomic DNA using PCR. Mouse strains utilized in this project were bred and housed in the mouse facility at the University of Texas Southwestern Medical Center in a specific pathogen-free environment following animal protocols that received approval from the Institutional Animal Care and Use Committee.

### Flow Cytometry and Sorting.

For surface marker staining, splenocytes were labeled with antibodies carrying fluorescent tags and then immersed in 2% (wt/vol) paraformaldehyde (Electron Microscopy Sciences) for fixation of cells before flow cytometric analysis. For detection of intracellular IFN-γ expression, splenocytes were treated with Brefeldin A (eBioscience) and PMA plus ionomycin for 4 h. Cells were then permeabilized with 0.1% saponin solution and labeled with antibodies. A FACSCalibur machine (BD Biosciences) and FlowJo software were utilized to run the analysis. For CD11c^+^ cell isolation, splenocytes were processed with mouse CD11c MicroBeads UltraPure (Miltenyi Biotec) and MACS columns (Miltenyi Biotec). Subpopulations of CD11c^+^ cells were then further isolated by the FACSAria machine (BD Biosciences) after staining with antibodies. Antibodies utilized for flow cytometry analysis and cell sorting include the following: CD3-APC, CD44-FITC, CD62L-PE, CD8-PerCP, CD8-Alex488, CD4-APC, CD4-PE, CD69-PE-cy5, ly6c-APC, IFNγ-PE, CD11c-PE, CD11b-PerCP, and I-A/I-E-FITC.

### Western Blotting and ELISA.

For western blotting, cell lysis was performed in 2x sample buffer; samples were separated via electrophoresis using an SDS-PAGE gel (Biorad) and blotted with antibodies detecting cGAS (Cell signaling) and GAPDH (Cell signaling), followed by staining with secondary antibodies conjugated with HRP (Cell signaling). Anti-ssDNA and anti-dsDNA IgG levels were measured by ELISA. For ELISA analysis, 10 μg/ml of calf thymus ssDNA (Sigma) or 10 μg/ml of dsDNA (Adi) were incubated overnight in 96-well plates (Greiner Bio-One) at 4 °C. Plates were blocked with 10% FBS and incubated with sera (1:50 dilution) before readout using a goat anti-mouse IgG antibody conjugated with HRP (Invitrogen). OD at 450 nm was measured after providing the plates with the substrate 3,3',5,5'-tetramethylbenzidine (Thermo Scientific).

### Quantitative Reverse Transcriptase-PCR.

TRIzol Reagent (Invitrogen) was utilized to extract RNA from homogenized mouse heart or spleen samples. Per the manufacturer’s instructions, the cDNA reverse transcription kit (Applied Biosystems) was used to generate the cDNA which was then quantified using SYBR green master mix (Applied Biosystems). *SI Appendix*, Table S1 lists the primer sequences used for these procedures.

### Pathology.

First, 4% (wt/vol) paraformaldehyde was utilized to fix the tissues of interest. Samples were then embedded in paraffin and sectioned into 5-μm pieces before undergoing hematoxylin and eosin (H&E) staining for imaging and analysis. Heart tissues were processed with H&E and Picro-Sirius red staining solutions. Histological scores were measured by combining the inflammation and fibrosis scores as described previously (23).

### Statistics.

Mouse survival curves were analyzed via the Mantel–Cox test. Unless otherwise noted, a two-tailed, unpaired Student’s t test was used to perform statistical analyses.

## Supplementary Material

Appendix 01 (PDF)

## Data Availability

All study data are included in the article and/or *SI Appendix*.
