# Supplementary material for: cGAS activation in classical dendritic cells causes autoimmunity in TREX1-deficient mice
Source: Proc Natl Acad Sci U S A. 2024 Sep 10;121(38):e2411747121. doi: 10.1073/pnas.2411747121 (PMC11420187; doi:10.1073/pnas.2411747121)
Supplement: Supplementary file 1 — Appendix 01 (PDF) [file pnas.2411747121.sapp.pdf]

PNAS

Supporting Information for
cGAS activation in classical dendritic cells causes autoimmunity in TREX1-deficient mice

Tong Li, Seoyun Yum, Junjiao Wu, Minghao Li, Yafang Deng, Lijun Sun, Xiaoxia Zuo, and Zhijian J. Chen

Zhijian J. Chen
Email: Zhijian.chen@utsouthwestern.edu

This PDF file includes:

Figures S1 to S6
Table S1

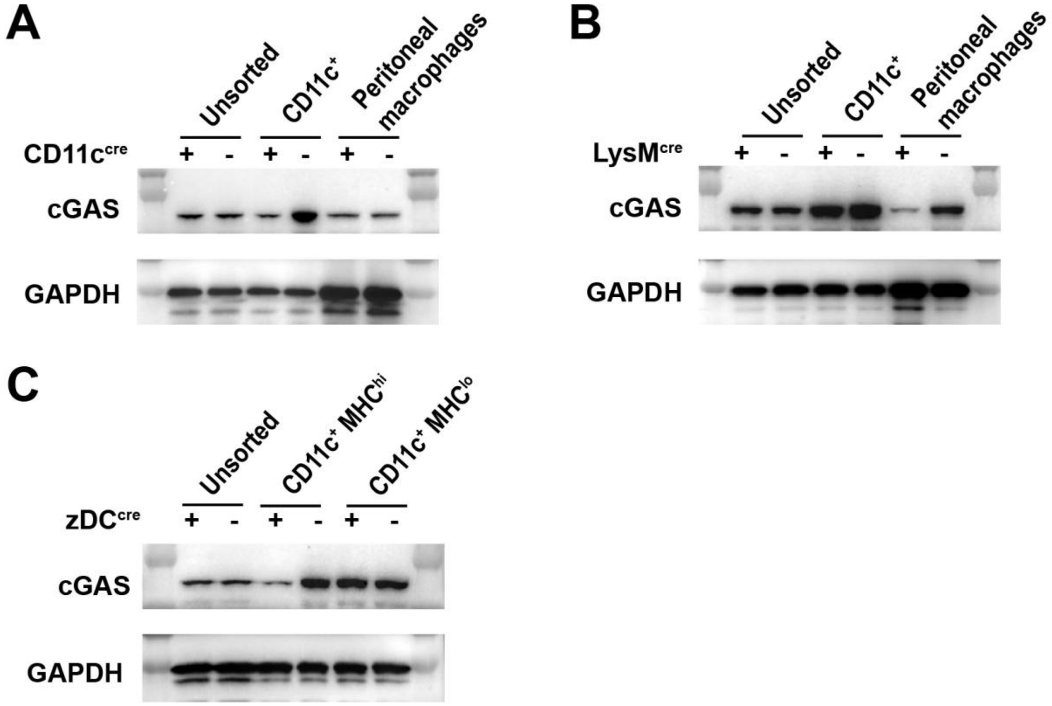

Fig. S1. cGAS expression in Trex1$^{-/-}$ cGAS-conditional KO mice. Western blots of cells isolated from Trex1$^{-/-}$ CD11c^{cre+} cGAS$^{Flox/Flox}$ (*A*), Trex1$^{-/-}$ LysM^{cre+} cGAS$^{Flox/Flox}$ (*B*), Trex1$^{-/-}$ zDC^{cre+} cGAS$^{Flox/Flox}$ (*C*), and their Cre-negative littermates. Unsorted (Splenocytes); CD11c^{+} (Splenic DCs); Peritoneal macrophages (Peritoneal cavity cells); CD11c^{+} MHC^{hi} (cDCs); CD11c^{+} MHC^{lo} (pDCs).

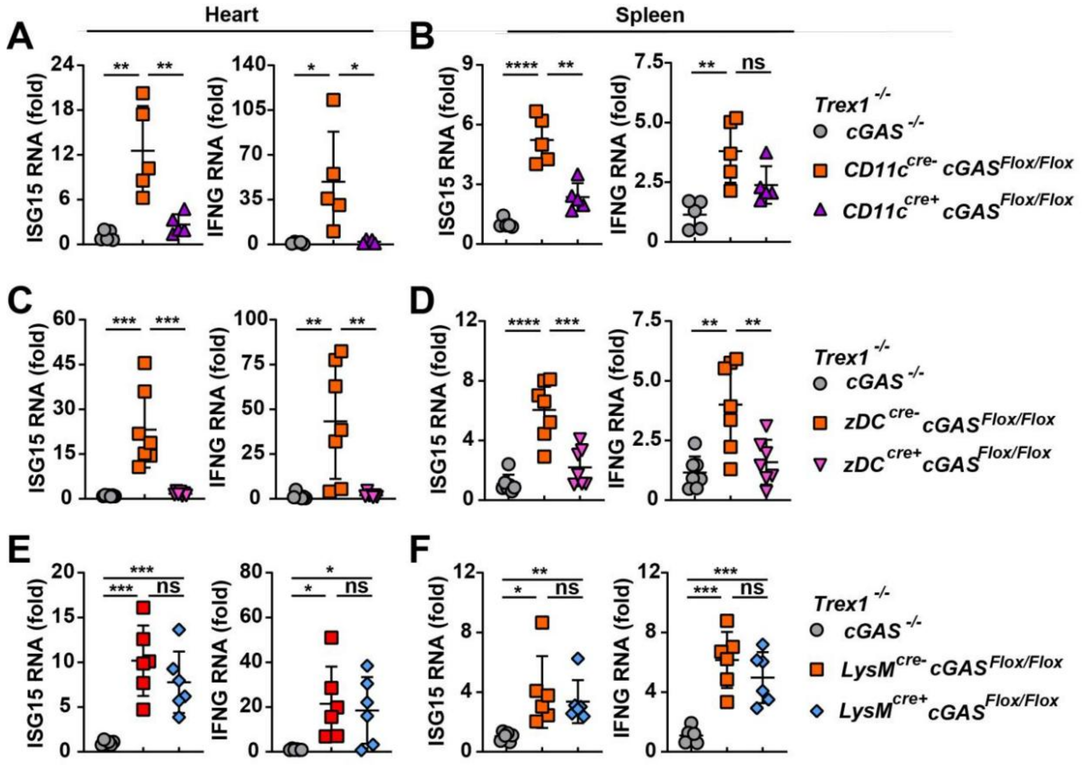

Fig. S2. cGAS in cDCs, but not in macrophages, is essential for the expression of ISGs in Trex1$^{-/-}$cGAS$^{Flox/Flox}$ mice. qRT-PCR analysis of indicated ISGs in the hearts (*A*, *C*, *E*) and spleens (*B*, *D*, *F*) from 3-month-old mice of indicated genotypes. Fold changes are relative to Trex1$^{-/-}$ cGAS$^{-/-}$ mice. Error bars represent SD. *p<0.05, **p<0.01, ***p<0.001, ****p<0.0001.

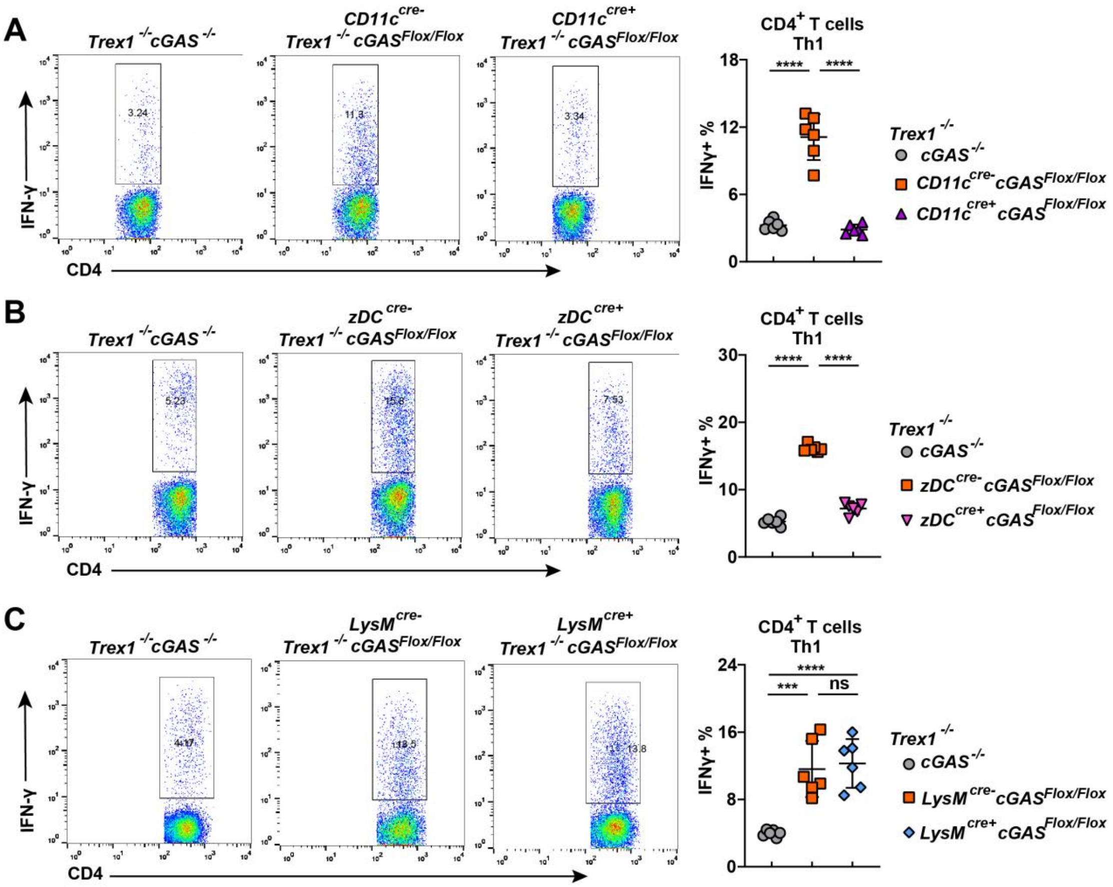

Fig. S3. cGAS in cDCs is required for the generation of Th1 cells in Trex1$^{-/-}$ cGAS$^{Flox/Flox}$ mice. Flow-cytometric analysis of intracellular IFN-γ expression after PMA plus ionomycin treatment in splenic CD4^{+} T cells from 3-month-old mice of the indicated genotypes. Error bars represent SD. **p*<0.05, ***p*<0.01, ****p*<0.001, *****p*<0.0001.

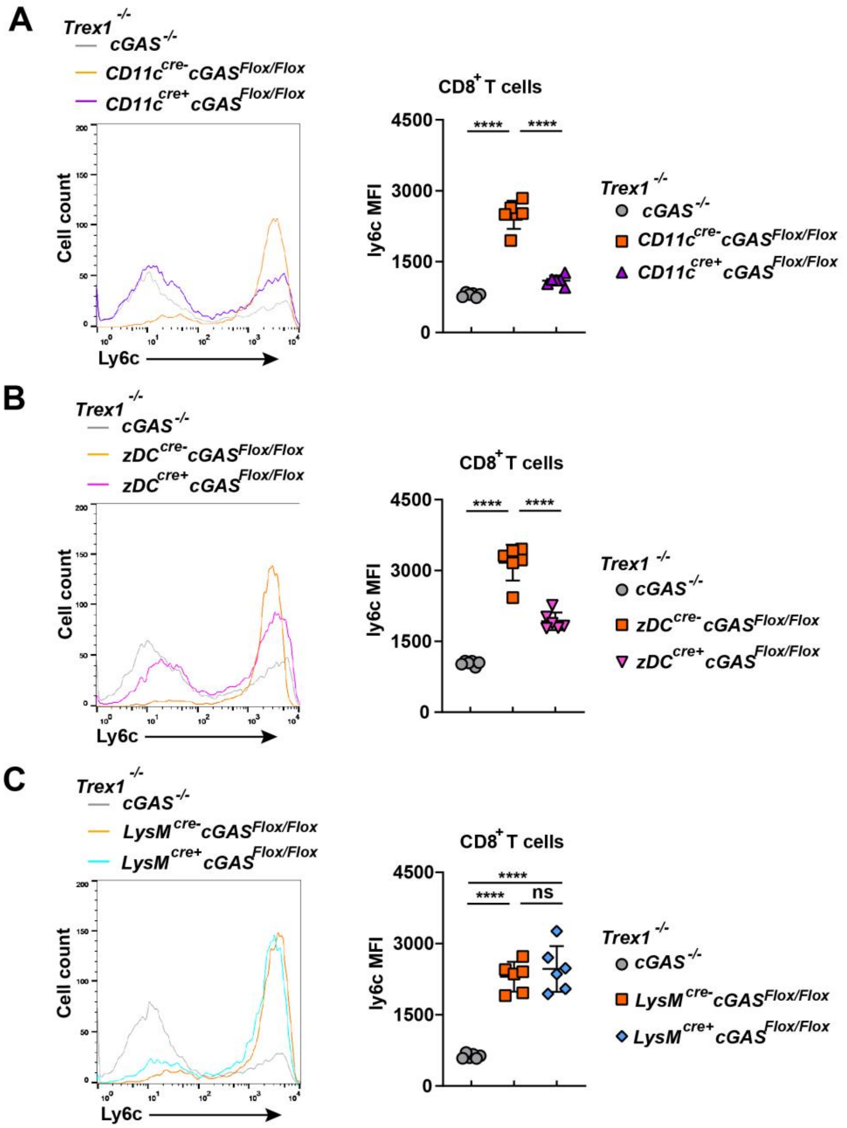

Fig. S4. cGAS in cDCs is required for the generation of hyperreactive CD8$^+$ T cells in Trex1$^{-/-}$ cGAS$^{Flox/Flox}$ mice. Flow-cytometric analysis of surface Ly6c levels on splenic CD8$^+$ T cells from 3-month-old mice of the indicated genotypes. Error bars represent SD.*****p*<0.0001.

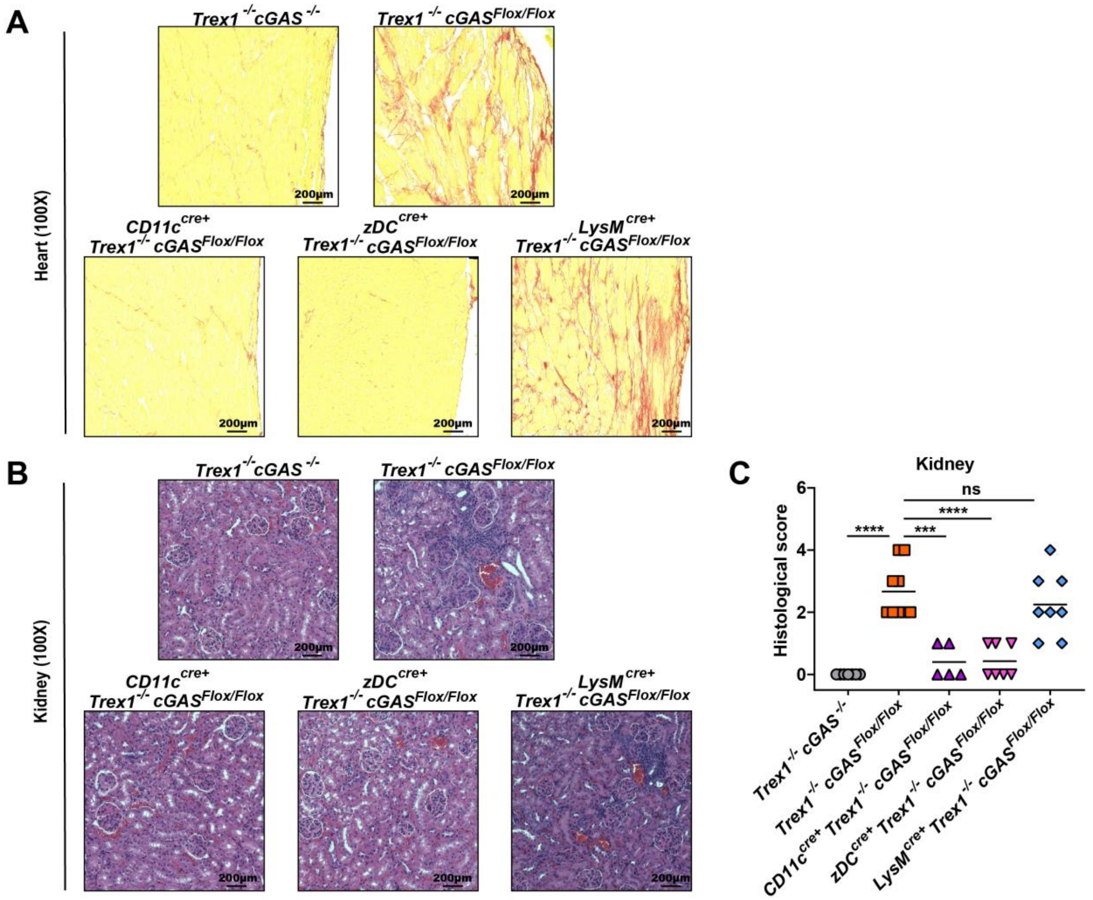

Fig. S5. cGAS in cDCs, but not in macrophages, mediates multiorgan inflammation in Trex1$^{-/-}$ cGAS$^{Flox/Flox}$ mice. (*A*) Representative Pico-Sirius Red (PSR; to measure fibrosis)-stained heart sections from 6-month-old mice of the indicated genotypes. (*B*) Representative H&E-stained kidney sections from 6-month-old mice of the indicated genotypes. (*C*) Blinded analysis of the kidney sections. Histological scores were calculated as described in Material and Methods. Statistical analysis was performed with a two-tailed, unpaired Student's t-test. ***p<0.001, **** p<0.0001.

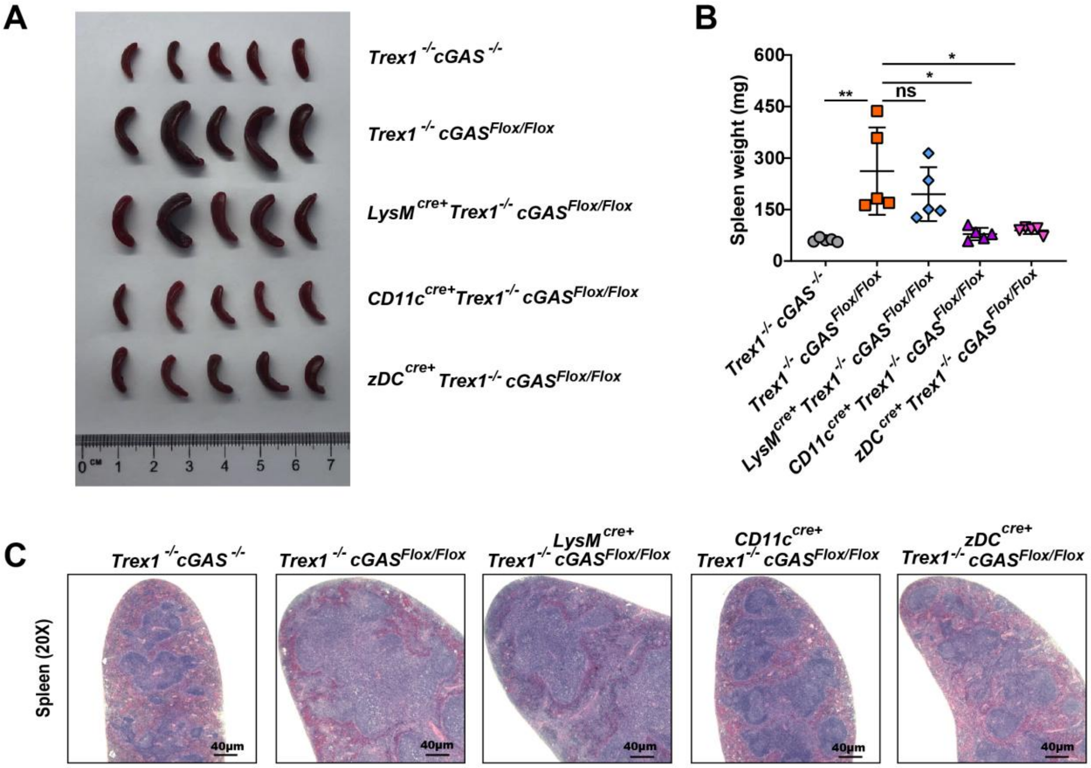

Fig. S6. cGAS in cDCs, but not in macrophages, is responsible for the splenomegaly of Trex1$^{-/-}$ cGAS$^{Flox/Flox}$ mice. (A) Spleens from 6-month-old mice of the indicated genotypes. (B) Spleen weights. (C) Representative H&E-stained spleen sections. Error bars represent SD. **p*<0.05, ***p*<0.01.

Table S1. qPCR primers

Gene	Forward (5’ to 3’)	Reverse (5’ to 3’)
Rpl19	AAATCGCCAATGCCAACTC	TCTTCCCTATGCCCATATGC
Cxcl10	GCCGTCATTTTCTGCCTCA	CGTCCTTGCGAGAGGGATC
Ifit3	TGGCCTACATAAAGCACCTAGATGG	CGCAAACTTTTGGCAAACTTGTCT
Irf7	ATGCACAGATCTTCAAGGCCTGGGC	GTGCTGTGGAGTGCACAGCGGAAGT
Isg15	GGAACGAAAGGGGCCACAGCA	CCTCCATGGGCCTTCCCTCGA
Ifng	CTTTGCAGCTCTTCCTCATGGCTGTTTCTG	TGACGCTTATGTTGTTGCTGATGGCCTG
